# Involvement of AMPK in Alcohol Dehydrogenase Accentuated Myocardial Dysfunction Following Acute Ethanol Challenge in Mice

**DOI:** 10.1371/journal.pone.0011268

**Published:** 2010-06-23

**Authors:** Rui Guo, Glenda I. Scott, Jun Ren

**Affiliations:** Center for Cardiovascular Research and Alternative Medicine, College of Health Sciences, University of Wyoming, Laramie, Wyoming, United States of America; Ohio State University, United States of America

## Abstract

**Objectives:**

Binge alcohol drinking often triggers myocardial contractile dysfunction although the underlying mechanism is not fully clear. This study was designed to examine the impact of cardiac-specific overexpression of alcohol dehydrogenase (ADH) on ethanol-induced change in cardiac contractile function, intracellular Ca^2+^ homeostasis, insulin and AMP-dependent kinase (AMPK) signaling.

**Methods:**

ADH transgenic and wild-type FVB mice were acutely challenged with ethanol (3 g/kg/d, i.p.) for 3 days. Oral glucose tolerance test, cardiac AMP/ATP levels, cardiac contractile function, intracellular Ca^2+^ handling and AMPK signaling (including ACC and LKB1) were examined.

**Results:**

Ethanol exposure led to glucose intolerance, elevated plasma insulin, compromised cardiac contractile and intracellular Ca^2+^ properties, downregulated protein phosphatase PP2A subunit and PPAR-γ, as well as phosphorylation of AMPK, ACC and LKB1, all of which except plasma insulin were overtly accentuated by ADH transgene. Interestingly, myocardium from ethanol-treated FVB mice displayed enhanced expression of PP2Cα and PGC-1α, decreased insulin receptor expression as well as unchanged expression of Glut4, the response of which was unaffected by ADH. Cardiac AMP-to-ATP ratio was significantly enhanced by ethanol exposure with a more pronounced increase in ADH mice. In addition, the AMPK inhibitor compound C (10 µM) abrogated acute ethanol exposure-elicited cardiomyocyte mechanical dysfunction.

**Conclusions:**

In summary, these data suggest that the ADH transgene exacerbated acute ethanol toxicity-induced myocardial contractile dysfunction, intracellular Ca^2+^ mishandling and glucose intolerance, indicating a role of ADH in acute ethanol toxicity-induced cardiac dysfunction possibly related to altered cellular fuel AMPK signaling cascade.

## Introduction

Episodic excessive alcohol consumption or binge drinking is a devastating public health problem associated with not only somatic complications but also traffic accidents, violent behavior and suicide [1]. Binge drinking-associated acute alcohol (ethanol) toxicity is often accompanied with a variety of risks including compromised myocardial contractile function [1], [2]. Although a number of theories have been put forward regarding the onset and progression of ethanol toxicity-induced myopathic changes including toxicity of ethanol and its metabolites, oxidative stress and accumulation of fatty acid ethyl esters [3], [4], the precise mechanism(s) of action behind alcohol-elicited cardiac damage remains controversial. Data from our lab have suggested that acetaldehyde, the primary metabolic product of ethanol, may contribute to the ethanol-induced cardiac damage by compromising myocardial excitation-contraction coupling, sarco(endo)plasmic reticulum (SR) Ca^2+^ release and cardiac contractile function [5], [6], [7]. This notion received further support from our recent observations of lessened cardiac morphological and functional damage with the facilitated clearance of acetaldehyde via mitochondrial aldehyde dehydrogenase (ALDH-2) following acute or chronic ethanol exposure [8], [9]. More interestingly, evidence also depicted a role of energy metabolism in the ethanol-elicited tissue and cell damage as ethanol ingestion has been shown to reduce the level or activity of ATP, cytochrome oxidase, succinate dehydrogenase and NADH dehydrogenase as well as decrease mitochondrial respiratory rate and phosphorylation efficiency in a variety of tissues such as heart, brain and stomach [10], [11], [12]. This is somewhat coordinated with our earlier finding that alcohol dehydrogenase (ADH), which oxidizes ethanol into acetaldehyde, exacerbated mitochondrial dysfunction manifested as loss of mitochondrial membrane potential and accumulation of mitochondrial O_2_^−^ anion [13]. Given that cardiac energy metabolism is shown to be altered in response to ethanol intake [14] which may play a key role in alcoholism-elicited cardiac contractile function, our present study was designed to address the role of cellular fuel AMP-activated protein kinase (AMPK) cascade in acute ethanol exposure-induced myocardial dysfunction. We hereby took advantage of a transgenic mouse model with the cardiac-specific overexpression of ADH, which mimics an accelerated alcoholic cardiomyopathy model of alcoholic cardiomyopathy [15]. Cardiac contractile function, intracellular Ca^2+^ handling, cardiac AMP/ATP ratio, the main up- and down-stream signaling molecules of AMPK including LKB1 and acetyl-CoA carboxylase (ACC) were examined. Expression of proteins closely associated with energy metabolism and insulin signaling including PPARγ, PGC-1α, insulin receptor β and Glut4 was examined in wild-type FVB and ADH hearts following acute ethanol challenge. To monitor the change in the AMPK degrading phosphatase, expression of protein phosphatase 2A (PP2A) and protein phosphatase 2Cα (PP2Cα) was examined. Oral glucose tolerance test and plasma insulin levels were measured for the overall assessment of glucose handling capacity.

## Materials and Methods

### Experimental animals and acute ethanol exposure

All animal procedures described here were in accordance with humane animal care standards outlined in the NIH Guide for the Care and Use of Experimental and were approved the University of Wyoming Animal Care and Use Committee (#A-3216-01). Production of the ADH transgenic mice was described in detail previously [16]. In brief, using the albino Friend Virus-B type (FVB) mice, the cDNA for murine class I ADH was inserted behind mouse α-myosin heavy chain promoter to achieve cardiac-specific overexpression. This cDNA was chosen because class I ADH is the most efficient in the oxidation of ethanol. A second transgene with a cDNA encoding tyrosinase was co-injected with ADH. This enzyme produces coat color pigmentation in albino mice and was used to conveniently identify transgenic animals. All mice were housed in a temperature-controlled room under a 12 hr/12 hr-light/dark and allowed access to tap water *ad libitum*. For acute ethanol challenge, adult male FVB and ADH mice (5–7 month-old) were injected intraperitoneally with ethanol (3 g/kg/d) for 3 consecutive days [13] prior to euthanasia under anesthesia (ketamine/xylazine: 3∶1, 1.32 mg/kg, i.p.) 72 hrs after the initial ethanol injection. Six hours after ethanol or saline challenge, blood samples were taken from the tail vein and immediately deproteinized with 6.25% trichloroacetic acid solution. Plasma insulin levels were measured using an ELISA commercial kit.

### Oral glucose tolerance test (OGTT)

Following a 12-hr overnight fasting period, FVB and ADH mice with or without acute ethanol challenge were gavaged with glucose (2 g/kg b.w.). Blood samples were drawn from the tail vein immediately before the glucose challenge, as well as 15, 30, 60, 90 and 120 min thereafter. Serum glucose levels were determined using an Accu-Chek III glucose analyzer. The area under the curve (AUC) was calculated using trapezoidal analysis for each adjacent time point and serum glucose level [17].

### Assessment of ethanol level

Upon sacrifice under anesthesia (ketamine/xylazine: 3∶1, 1.32 mg/kg, i.p.), plasma was collected and was stored in sealed vials at −80°C. Immediately before analysis, samples were warmed to 25°C. A 2 ml aliquot of the headspace gas from each vial was removed through the septum on the cap with a gas tight syringe and transferred to a 200 µl loop injection system on a Hewlett-Packard 5890 gas chromatograph (GC) equipped with a flame ionization detector. Ethanol was separated on a 9-meter VOCOL capillary column (Supelco) with film with 1.8 µm thickness and an inner diameter of 0.32 mm. The temperature was held isothermally at 30°C, and the carrier gas was helium at a flow rate of 1.8 ml/min. Quantitation was achieved by calibrating the GC peak areas against those from headspace samples of known ethanol standards, over a similar concentration range as the tissue samples in the same buffer [16].

### HPLC assay of AMP/ATP content

The heart tissues were extracted by 6% perchloric acid (Sigma, St. Louis, MO). The acidic homogenate was kept on ice for 30 min, and then centrifuged at 14,000 rpm at 4°C for 10 min. An aliquot of the pellets was set aside for protein measurements. The supernatant was neutralized with 1 mol/l K_2_CO_3_, adjust pH to 3.5. Then kept the supernatant on ice for 10 min and at −80°C for 1–2 h to promote precipitation of the perchlorate and centrifuged again. Supernatants were stored at −80°C until HPLC assay. The chromatographic separation of AMP was performed using a Grace Partisil SAX column (250 mm ×4 mm i.d., particle size 10 µm) (Deerfield, IL). The mobile phases were composed of a gradient of 5 mM ammonium dihydrogen phosphate (pH 2.8) and 750 mM ammonium dihydrogen phosphate (pH 3.9). The flow rate was varied from1–2 ml/min over the course of the gradient profile to provide a reasonable assay time of 25 min. The sample injection volume was 50 µl and the components were monitored at 254 nm. The Beckman GOLD HPLC system was operated in laboratory at room temperature (23–25°C). Concentrations were determined by construction of a calibration curve range from 1 to 80 nmoles per 50 µl injected. Standard stock solutions for calibration curve construction were 6.4 µmole/ml AMP and ATP prepared in 5 mmol/l ammonium dihydrogen phosphate (pH 2.8). These solutions were stored at −80°C and used as references for peaks quantification. Fresh dilution was made before each assay to construct a calibration curve, adding 5 mmol/l ammonium dihydrogen phosphate (pH 2.8) in order to obtain 1, 5, 10, 20, 40 and 80 nmoles per 50 µ l injected [18].

### Isolation of murine cardiomyocytes

After ketamine/xylazine sedation, hearts were removed and perfused with Krebs-Henseleit bicarbonate (KHB) buffer containing (in mM): 118 NaCl, 4.7 KCl, 1.2 MgSO_4_, 1.2 KH_2_PO_4_, 25 NaHCO_3_, 10 HEPES and 11.1 glucose. Hearts were digested with collagenase D for 20 min. Left ventricles were removed and minced before being filtered. Myocyte yield was 50%–70% which was not overtly affected by ADH or ethanol challenge [13]. To directly assess the role of AMPK in acute ethanol exposure-induced cardiomyocyte contractile response, cardiomyocytes from adult wild-type FVB mice were treated with ethanol (240 mg/dl) at 37°C for 2 hrs in the absence or presence of the AMPK inhibitor compound C (10 µM) [19] before mechanical function was assessed.

### Cell shortening/relengthening

Mechanical properties of cardiomyocytes were assessed using a SoftEdge MyoCam system (IonOptix Corporation, Milton, MA) [18]. In brief, cardiomyocytes were placed in a chamber mounted on the stage of an inverted microscope (Olympus, IX-70) and superfused at 25°C with a buffer containing (in mM): 131 NaCl, 4 KCl, 1 CaCl_2_, 1 MgCl_2_, 10 Glucose and 10 HEPES, at pH 7.4. The cells were field stimulated with suprathreshold voltage at a frequency of 0.5 Hz, 3 msec duration, using a pair of platinum wires placed on opposite sides of the chamber and connected to an electrical stimulator (FHC Inc, Brunswick, NE). The myocyte being studied was displayed on a computer monitor using an IonOptix MyoCam camera. An IonOptix SoftEdge software was used to capture changes in cell length during shortening and relengthening. Cell shortening and relengthening were assessed using the following indices: peak shortening (PS), maximal velocities of cell shortening and relengthening (± dL/dt), time-to-PS (TPS), and time-to-90% relengthening (TR_90_). In the case of altering stimulus frequency from 0.1 to 5.0 Hz, the steady state contraction of myocyte was achieved (usually after the first 5–6 beats) before PS was recorded.

### Intracellular Ca^2+^ fluorescence measurement

Myocytes were loaded with fura-2/AM (0.5 µM) for 10 min and fluorescence measurements were recorded with a dual-excitation fluorescence photo multiplier system (Ionoptix). Cardiomyocytes were placed on an Olympus IX-70 inverted microscope and imaged through a Fluor 40× oil objective. Cells were exposed to light emitted by a 75 W lamp and passed through either a 360 or a 380 nm filter, while being stimulated to contract at 0.5 Hz. Fluorescence emissions were detected between 480 and 520 nm by a photomultiplier tube after first illuminating the cells at 360 nm for 0.5 s then at 380 nm for the duration of the recording protocol (333 Hz sampling rate). The 360 nm excitation scan was repeated at the end of the protocol and qualitative changes in intracellular Ca^2+^ concentration were inferred from the ratio of fura-2 fluorescence intensity at two wavelengths (360/380). Fluorescence decay time was assessed as an indication of intracellular Ca^2+^ clearing. Both single and bi-exponential curve fits were applied to calculate the intracellular Ca^2+^ decay constant [18].

### Mouse heart perfusion

Isolated mouse hearts were retrogradely perfused with a Krebs-Henseleit buffer containing 7 mM glucose, 0.4 mM oleate, 1% BSA and a low fasting concentration of insulin (10 µU/ml). Hearts were perfused at a constant flow of 4 ml/min (equal to an aortic pressure of 80 cmH_2_O) at baseline for 30 min to reach the steady-state. A fluid-filled latex balloon connected to a solid-state pressure transducer was inserted into the left ventricle through a left atriotomy to measure left ventricular pressure between 30 and 90 min after initiation of perfusion. LVDP and the first derivative of LVDP (± dP/dt) were recorded using a digital acquisition system at a balloon volume which resulted in a baseline LV end-diastolic pressure of 5 mmHg [13].

### Western blot analysis

Myocardial protein from the left ventricles was prepared as described [18]. Samples containing equal amount of proteins were separated on 10% SDS-polyacrylamide gels in a minigel apparatus (Mini-PROTEAN II, Bio-Rad) and transferred to nitrocellulose membranes. The membranes were blocked with 5% milk in TBS-T, and were incubated overnight at 4°C with anti-insulin receptor β, anti-PPAR-γ, anti-PGC1α, anti-Glut4, anti-AMPK, anti-phosphorylated AMPK (pAMPK, Thr172), anti-ACC, anti-phosphorylated ACC (pACC, Ser79), anti-LKB1, anti-phosphorylated LKB1 (pLKB1, Ser428), anti-PP2AA, anti-PP2AB, anti-PP2Cα and anti-GAPDH (loading control) antibodies. After washing blots to remove excessive primary antibody binding, blots were incubated for 1 hr with horseradish peroxidase (HRP)–conjugated secondary antibody (1∶5,000). Antibody binding was detected using enhanced chemiluminescence (Amersham Pharmacia, Piscataway, NJ), and film was scanned and the intensity of immunoblot bands was detected with a Bio-Rad Calibrated Densitometer (Model: GS-800).

## Data Analysis

Data are Mean ± SEM. Difference was calculated by repeated measures analysis of variance (ANOVA) followed by a Tukey's post hoc analysis. A p value <0.05 was considered significant.

## Results

### General features and OGTT of FVB and ADH mice treated with ethanol

**Figure 1:**
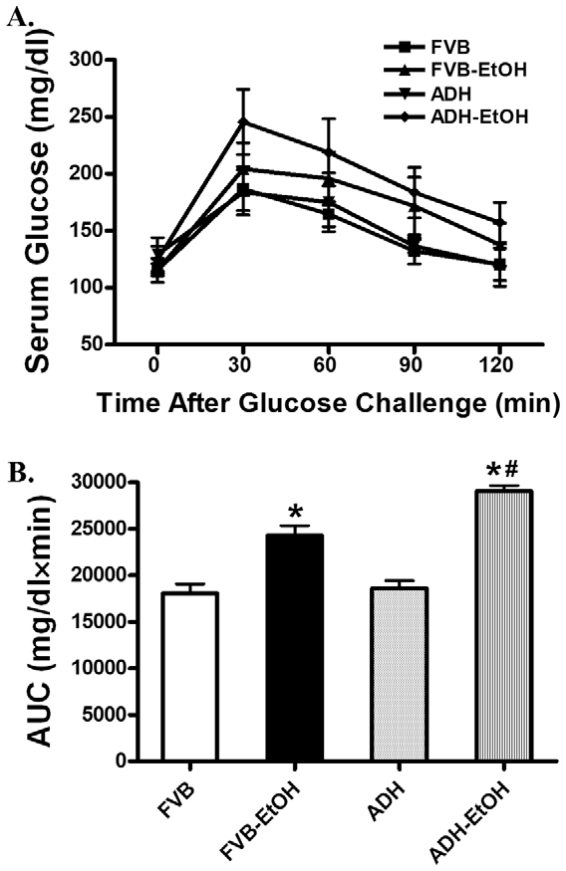
Oral glucose tolerance test (OGTT, 2 g/kg) in adult FVB and ADH mice with or without acute ethanol (EtOH) challenge (3 g/kg, i.p. for 3 days). A: Serum glucose levels within 120 min following acute glucose challenge; B: Area underneath the curve (AUC). Mean ± SEM, n = 7–9 mice per group, * p<0.05 *vs.* FVB group, # p<0.05 *vs.* FVB-EtOH group.

Neither ethanol treatment nor ADH transgene altered body and organ (heart, liver and kidney) weights or organ size (manifested as the organ-to-body weight ratio). As expected, acute ethanol exposure elicited comparable elevations in blood alcohol level compared with the non-ethanol-treated mice. While neither acute ethanol exposure nor ADH overtly affected cardiac ATP levels, acute ethanol treatment significantly elevated the AMP levels and AMP/ATP ratio in both mouse groups with a more pronounced increase in AMP/ATP ratio in the ADH mice. Plasma insulin levels and blood alcohol levels were significantly elevated in a comparable manner in both groups following acute ethanol challenge (Table 1). Following the oral glucose challenge, serum glucose levels in all four mouse groups began to drop after peaking at 15 min and returned towards baseline values after 120 min. Ethanol-treated FVB and ADH mice (FVB-EtOH and ADH-EtOH) both displayed slightly elevated (although non-significant) serum glucose levels between 30 and 90 min after glucose challenge compared with WT mice. This is consistent with the significantly greater area underneath the curve (AUC) in both ethanol-treated groups with an exaggeration in ADH-EtOH mice (Fig. 1). These data favor the existence of glucose intolerance following acute ethanol challenge with an exacerbated glucose disposal defect in ADH mice.

**Table 1:** Biometric and myocardial contractile parameters of FVB and ADH mice challenged with ethanol (3 g/kg, i.p. for 3 days).

Parameter	FVB	FVB-EtOH	ADH	ADH-EtOH
Body Weight (g)	26.6±0.6	26.6±0.9	27.0±0.6	27.0±0.7
Heart Weight (mg)	141±5	131±6	152±8	137±9
Heart/Body Weight (mg/g)	5.32±0.21	4.96±0.29	5.60±0.24	5.08±0.31
Liver Weight (g)	1.22±0.07	1.33±0.06	1.27±0.04	1.33±0.06
Liver/Body Weight (mg/g)	46.2±2.7	49.9±1.7	47.1±1.3	49.2±2.4
Kidney Weight (g)	0.32±0.02	0.33±0.02	0.31±0.01	0.36±0.02
Kidney/Body Weight (mg/g)	12.1±0.6	12.2±0.6	11.5±0.5	13.5±0.6
Cardiac AMP (nmol/mg protein)	7.64±0.27	24.35±6.72*	8.07±0.46	27.14±6.56*
Cardiac ATP (nmol/mg protein)	6.79±0.11	7.42±0.26	6.37±0.5	5.06±1.31
Cardiac AMP/ATP Ratio	1.13±0.04	3.36±0.84*	1.36±0.16	5.27±0.20*^,^ #
Plasma Insulin (ng/ml)	0.43±0.07	1.25±0.14*	0.49±0.07	1.29±0.17*
Blood Alcohol (mg/dl)	Undetectable	57.7±9.3*	Undetectable	75.2±19.5*

Mean ± SEM, n = 9–10 mice per group, undetectable: <2.5 mg/dl, * p<0.05 *vs.* FVB group,

^#^p<0.05 vs. FVB-EtOH group.

### Mechanical and intracellular Ca^2+^ properties of murine cardiomyocytes in FVB and ADH mice

**Figure 2:**
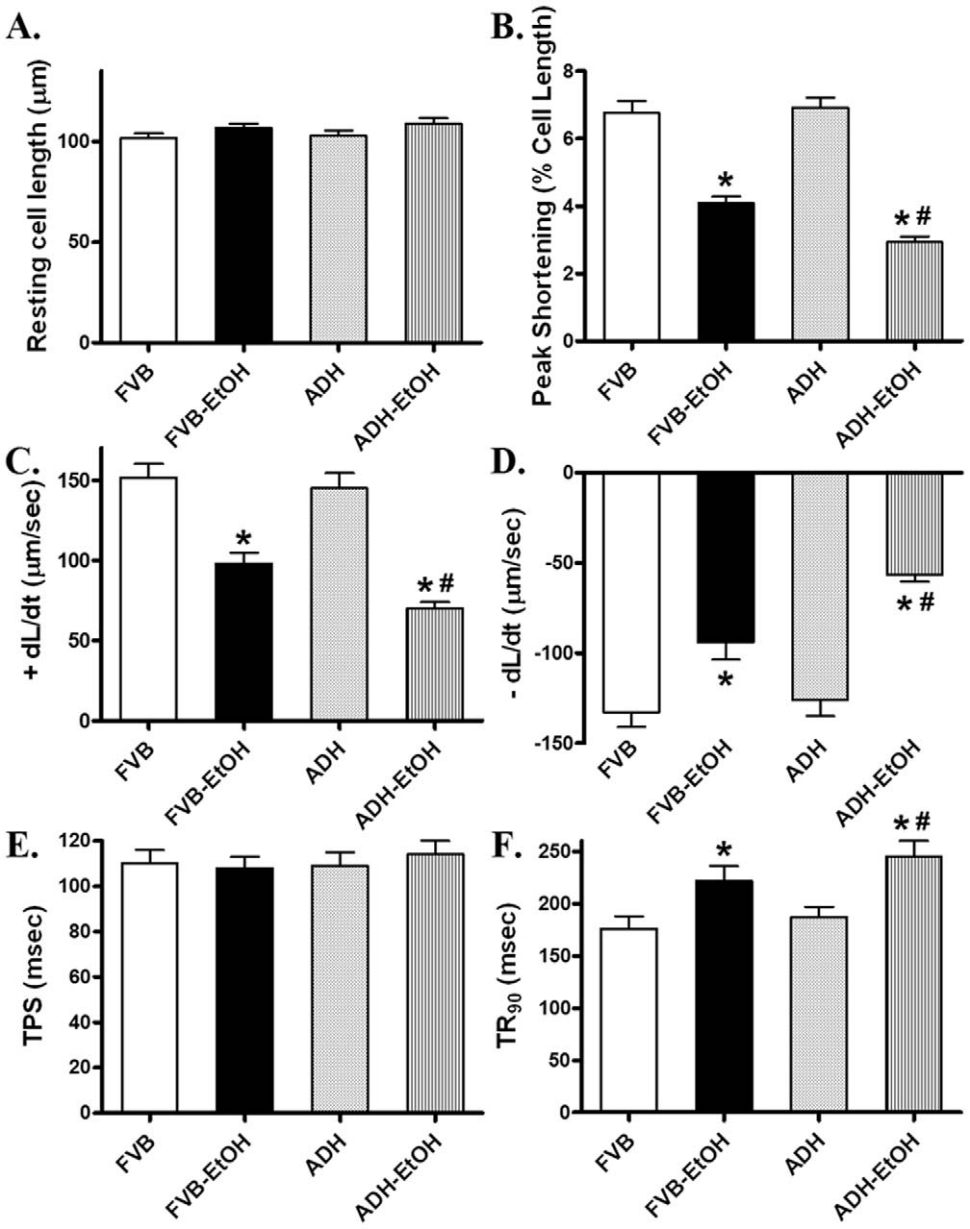
Cardiomyocyte contractile properties from adult FVB and ADH mice with or without acute ethanol (EtOH) challenge (3 g/kg, i.p. for 3 days). A: Resting cell length; B: Peak shortening (normalized to cell length); C: Maximal velocity of shortening (+ dL/dt); D: Maximal velocity of relengthening (− dL/dt); E: Time-to-PS (TPS); and F: Time-to-90% relengthening (TR_90_). Mean ± SEM, n = 80–81 cells from 3–4 mice per group, * p<0.05 *vs.*FVB group, # p<0.05 *vs.* FVB-EtOH group.

**Figure 3:**
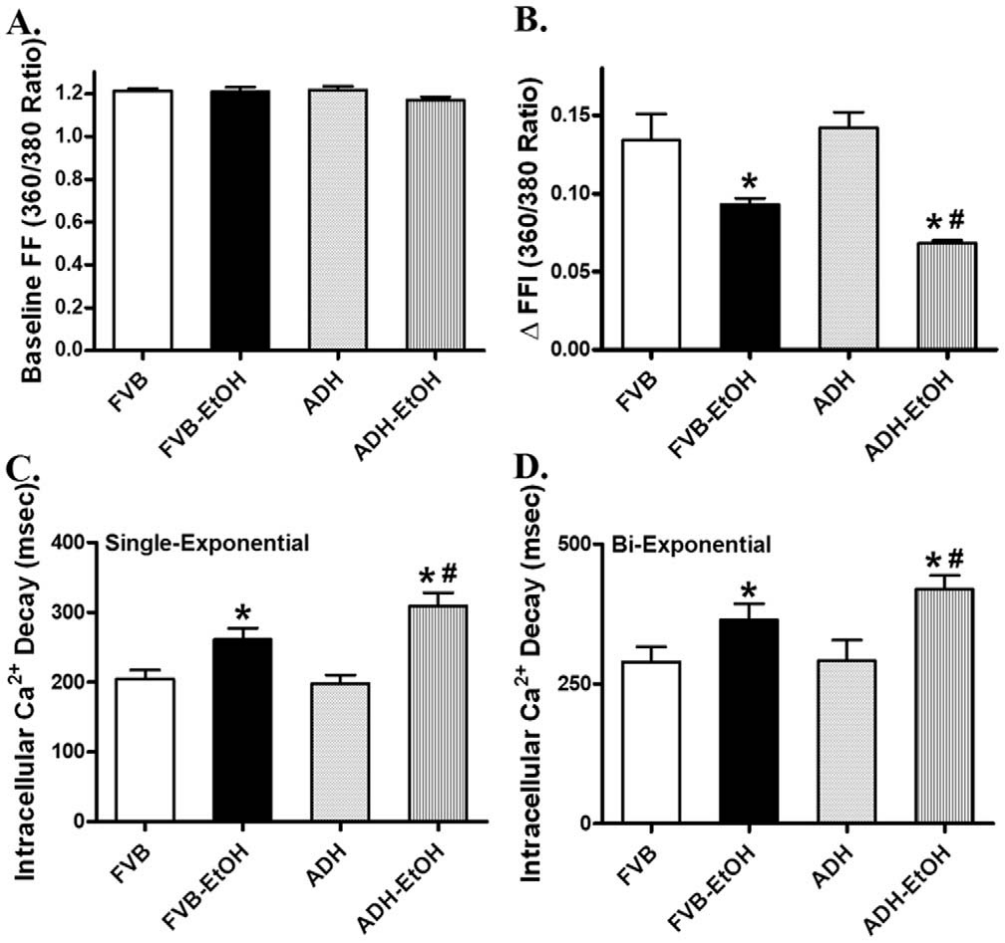
Intracellular Ca^2+^ transient properties in cardiomyocytes from adult FVB and ADH mice with or without acute ethanol (EtOH) challenge (3 g/kg, i.p. for 3 days). A: Baseline fura-2 fluorescence intensity (FFI); B: Electrically-stimulated rise in FFI (ΔFFI); C: Single exponential intracellular Ca^2+^ decay rate; and D: Bi-exponential intracellular Ca^2+^ decay rate. Mean ± SEM, n = 70 cells from 3–4 mice per group, * p<0.05 *vs.* FVB group, # p<0.05 *vs.* FVB-EtOH group.

Neither ethanol nor ADH transgene overtly affected resting cell length. However, cardiomyocytes from ethanol-treated FVB mice displayed significantly reduced peak shorting (PS) and maximal velocity of shortening/relengthening (± dL/dt) associated with prolonged time-to-90% relengthening (TR_90_) and normal time-to-PS (TPS). Consistent with previous finding from chronic alcohol administration study [15], ADH transgene accentuated the acute ethanol challenge-induced cardiomyocyte mechanical dysfunctions without eliciting any overt effect by itself (Fig. 2). To explore the potential mechanism(s) of action involved in the ADH-elicited augmentation of acute ethanol exposure-induced cardiomyocyte mechanical defect, intracellular Ca^2+^ homeostasis was evaluated using the fluorescence dye fura-2. Our results indicated that acute ethanol exposure significantly reduced electrically-stimulated rise in intracellular Ca^2+^ and prolonged intracellular Ca^2+^ decay rate (both single- and bi-exponential) without affecting basal intracellular Ca^2+^ levels, the effects of which were exacerbated by the ADH transgene. Consistent with the cell shortening response, ADH transgene failed to affect intracellular Ca^2+^ properties by itself (Fig. 3).

### Effect of increasing stimulation frequency on myocyte shortening

**Figure 4:**
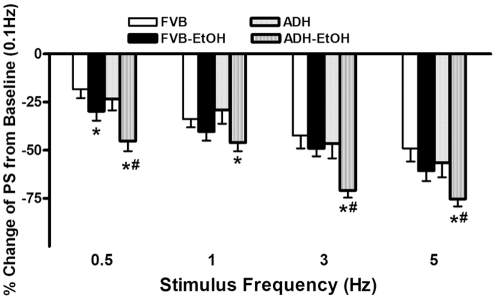
Change in cardiomyocyte contraction in response to increasing stimulus frequency (0.1–5.0 Hz) in adult FVB and ADH mice with or without acute ethanol (EtOH) challenge (3 g/kg, i.p. for 3 days). Peak shortening (PS) amplitude was normalized to the PS value obtained at 0.1 Hz from the same cell. Mean ± SEM, n = 24–26 cells from 3–4 mice per group, * p<0.05 *vs.*FVB group, # p<0.05 *vs.* FVB-EtOH group.

Murine hearts normally contract at high frequencies, whereas our mechanical recording was performed at 0.5 Hz. To evaluate the impact of acute ethanol exposure and/or ADH transgene overexpression on cardiac function under higher frequencies, we increased the stimulus frequency up to 5.0 Hz (300 beats/min) and recorded the steady-state peak shortening. Cardiomyocytes were initially stimulated to contract at 0.5 Hz for 5 min to ensure a steady-state before commencing the frequency response. Fig. 4 displays a negative staircase of peak shortening (PS) with increased stimulus frequency in all 4 groups with a steeper decline in PS in both ethanol-treated groups (although PS decline in FVB-EtOH group failed to reach significant level between 1 and 5 Hz). The loss in PS value was more pronounced in ethanol-treated ADH compared with FVB-EtOH group at all frequencies with the exception of 1 Hz. These data favor a possible role of reduced intracellular Ca^2+^ cycling or stress tolerance capacity under acute ethanol challenge, which may be accentuated by ADH.

### Effect of acute ethanol exposure on whole heart function in FVB and ADH mice

**Figure 5:**
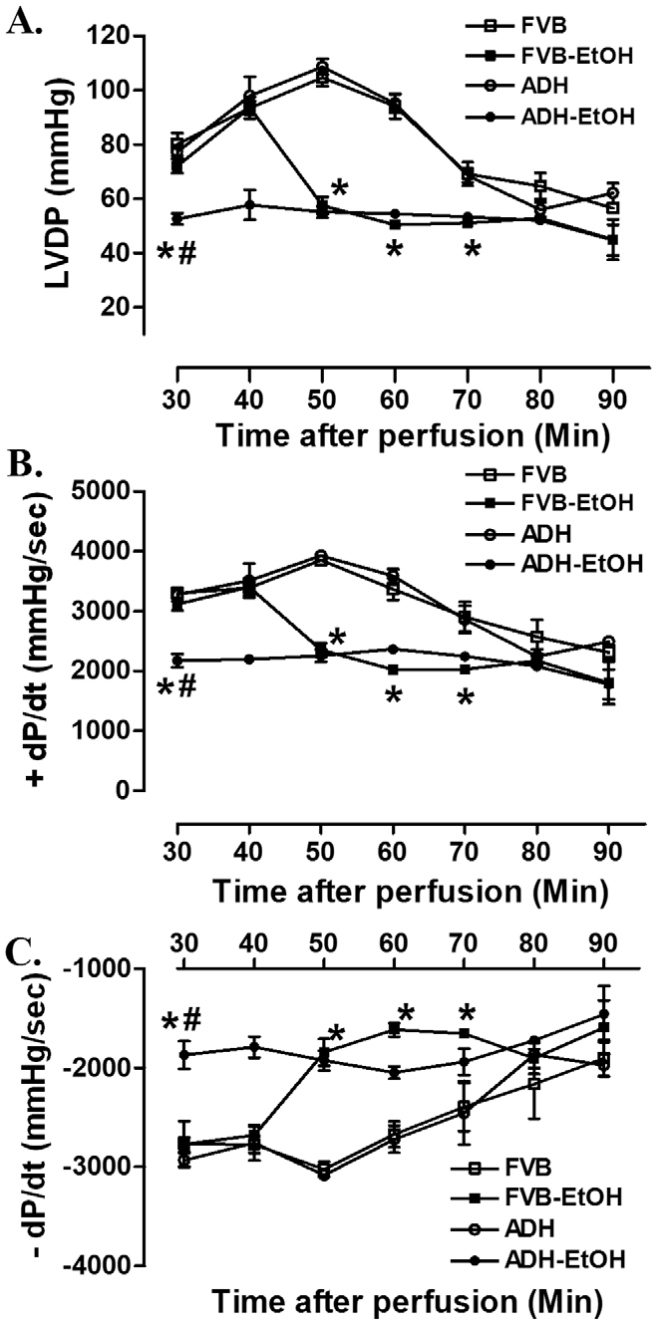
Langendorff myocardial contractile function from adult FVB and ADH mice with or without acute ethanol (EtOH) challenge (3 g/kg, i.p. for 3 days). Cardiac contractile function was assessed using a perfusion system for 90 min. A: Left ventricular developing pressure (LVDP); B and C: Maximal velocity of pressure development (+dP/dt) and decline (−dP/dt). Mean ± SEM, n = 4 hearts per group, * p<0.05 *vs.* FVB group, # p<0.05 *vs.* FVB-EtOH group.

To assess the impact ADH on myocardial contractile function in the setting of whole heart following acute ethanol challenge, the Langendroff perfused whole heart function was evaluated. Our data shown in Fig. 5 revealed that acute ethanol challenge resulted in a rapid decline in LVDP and ± dP/dt in FVB mice starting at 40 min of perfusion despite displaying normal baseline contractility parameters similar to those in FVB group. Interestingly, baseline LVDP and ± dP/dt were significantly lower in ADH mice treated with ethanol than FVB and FVB-EtOH groups and remained low throughout the 90 min perfusion duration. Overexpression of ADH transgene itself did not affect the baseline as well as time-dependent change in LVDP and ± dP/dt.

### Effect of acute ethanol challenge on insulin receptor, PPAR-γ, PGC1α and Glut4 in FVB and ADH mice

**Figure 6:**
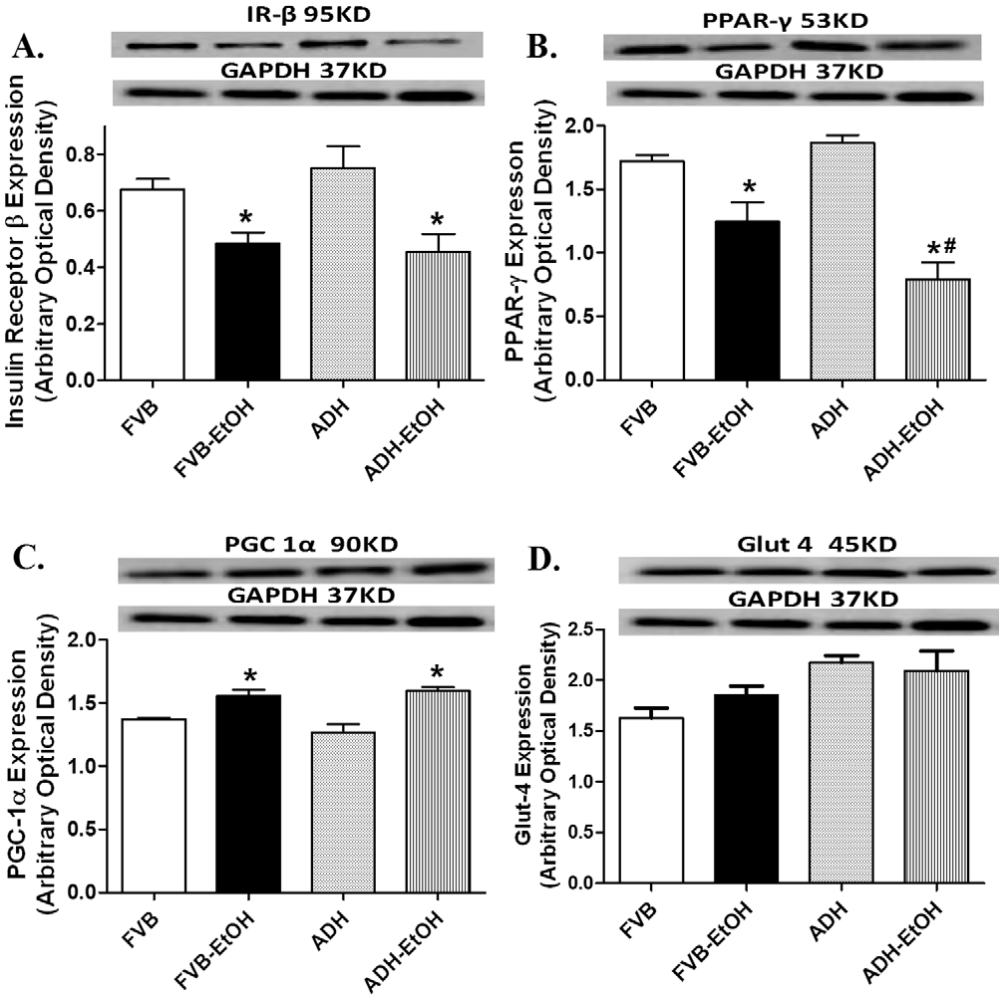
Expression of insulin receptor β (IR-β), PPAR-γ, PGC1α and Glut4 in myocardium from FVB and ADH mice with or without acute ethanol challenge (3 g/kg, i.p. for 3 days). A: IR-β; B: PPAR-γ; C: PGC1α and D: Glut4. Insets: Representative gel blots depicting expression of IR-β, PPAR-γ, PGC1α, Glut4 and GAPDH (loading control). Mean ± SEM, n = 6–8 samples per group, * p<0.05 *vs.* FVB group, # p<0.05 *vs*. FVB-EtOH group.

To better understand the ADH-induced exacerbation of the dampened glucose tolerance and myocardial contractile function in response to acute ethanol challenge, protein expression of insulin receptor, PPAR-γ, PGC1α and Glut4 was examined in FVB and ADH mice with or without ethanol challenge. Our data revealed that ethanol challenge downregulated insulin receptor β and upregulated PGC1α in a comparable manner in FVB and ADH groups without any effect from ADH transgene by itself. Consistent with the OGTT data, acute ethanol treatment significantly downregulated the insulin postreceptor signaling molecule PPAR-γ expression, the effect of which was exaggerated by ADH. Neither acute ethanol challenge nor ADH transgene affected expression of Glut4 (Fig. 6).

### Effect of acute ethanol challenge on AMPK, ACC and LKB1 in FVB and ADH mice

**Figure 7:**
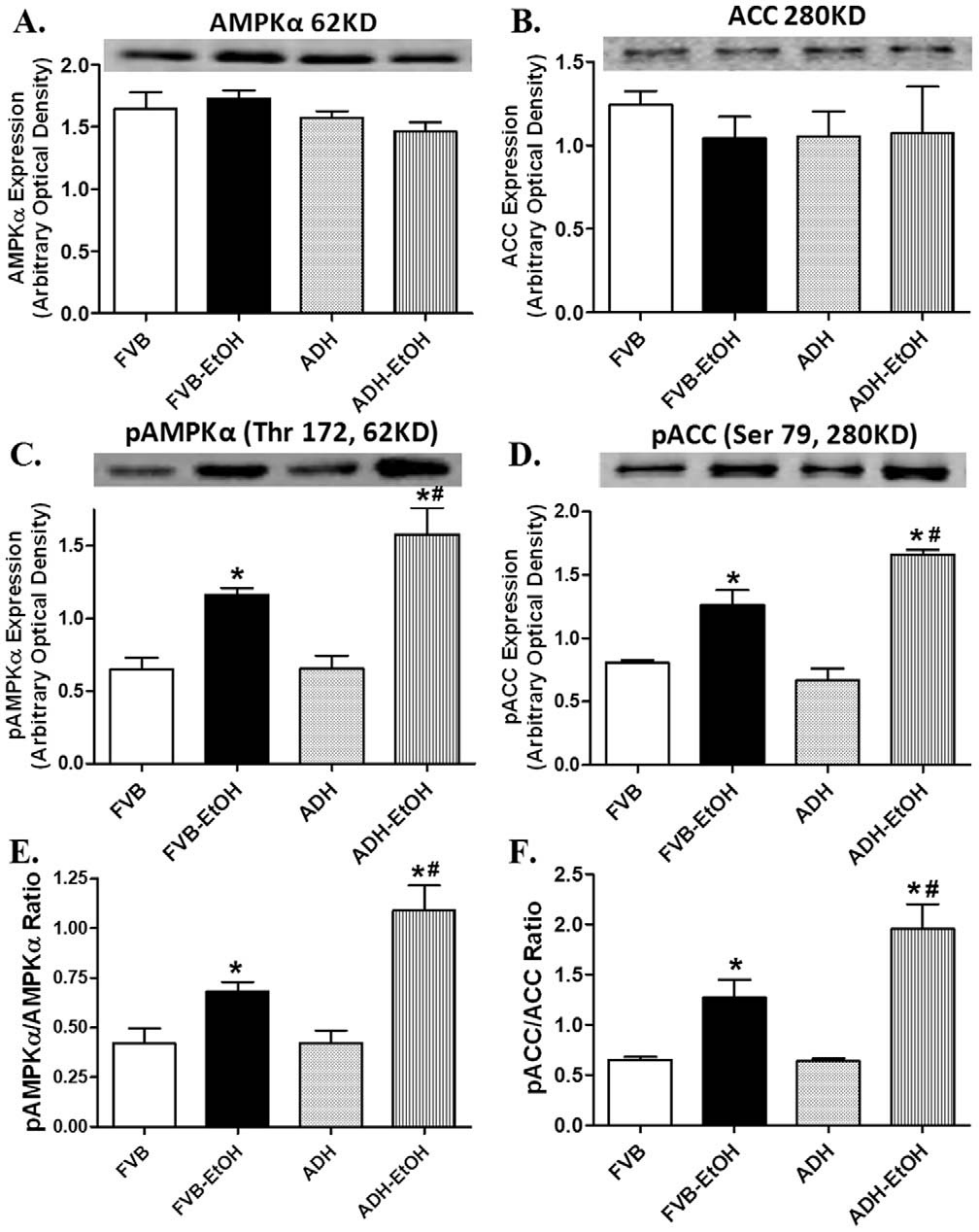
Expression of pan and phosphorylated AMPK and ACC in myocardium from FVB and ADH mice with or without acute ethanol challenge (3 g/kg, i.p. for 3 days). A: pan AMPK; B: pan ACC; C: phosphorylated AMPK (pAMPK); D: phosphorylated ACC (pACC); E: pAMPK/AMPK ratio and F: pACC/ACC ratio. Insets: Representative gel blots depicting expression of pan and phosphorylated AMPK and ACC. Mean ± SEM, n = 5–7 samples per group, * p<0.05 *vs.* FVB group, # p<0.05 *vs*. FVB-EtOH group.

**Figure 8:**
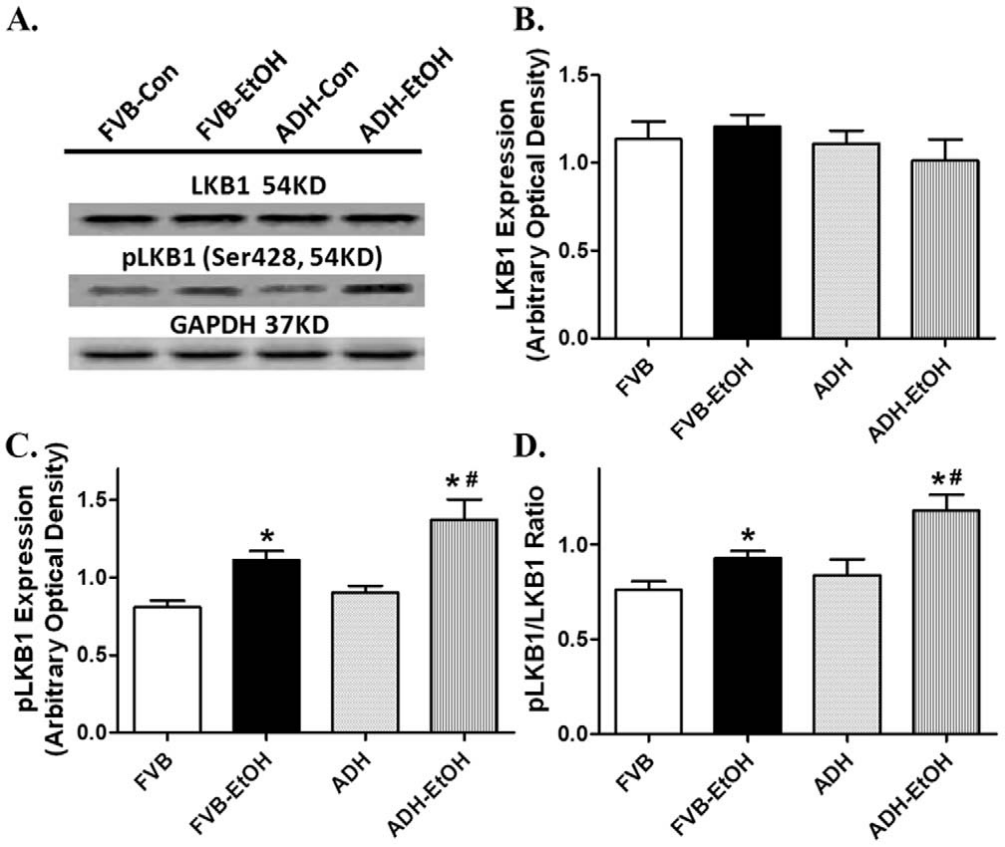
Expression of pan and phosphorylated LKB1 in myocardium from FVB and ADH mice with or without acute ethanol challenge (3 g/kg, i.p. for 3 days). A: Representative gel blots depicting expression of LKB1, phosphorylated LKB1 (pLKB) and GAPDH (loading control); B: pan LKB1; C: pLKB1; and D: pLKB1/LKB1 ratio. Mean ± SEM, n = 6–8 samples per group, * p<0.05 *vs.* FVB group, # p<0.05 *vs*. FVB-EtOH group.

To explore if the AMPK signaling cascade was involved in ADH and/or ethanol-induced cardiac contractile response, AMPK, its downstream signaling molecule ACC and the AMPK activating signal LKB1 were examined. Neither acute ethanol treatment nor ADH affected the expression of AMPK and ACC. However, phosphorylation of both AMPK and ACC was increased by acute ethanol exposure (both absolute value and phosphorylated-to-pan protein ratio), the effect of which was accentuated by ADH. ADH transgene itself did not elicit any effect on the phosphorylation of AMPK and ACC (Fig. 7). We went on to examine the involvement of LKB1 in ADH and ethanol-induced response in AMPK/ACC activation. Neither ethanol nor ADH transgene affected the levels of pan LKB1. In line with its effect on AMPK and ACC phosphorylation, acute ethanol challenge enhanced the phosphorylation of LKB1 (both absolute and pLKB1/LKB1 ratio), the effect of which was augmented by ADH transgene. Last but not the least, ADH transgene itself did not affect LKB1 phosphorylation (Fig. 8).

### Effect of acute ethanol challenge on protein phosphatase expression in FVB and ADH mice

**Figure 9:**
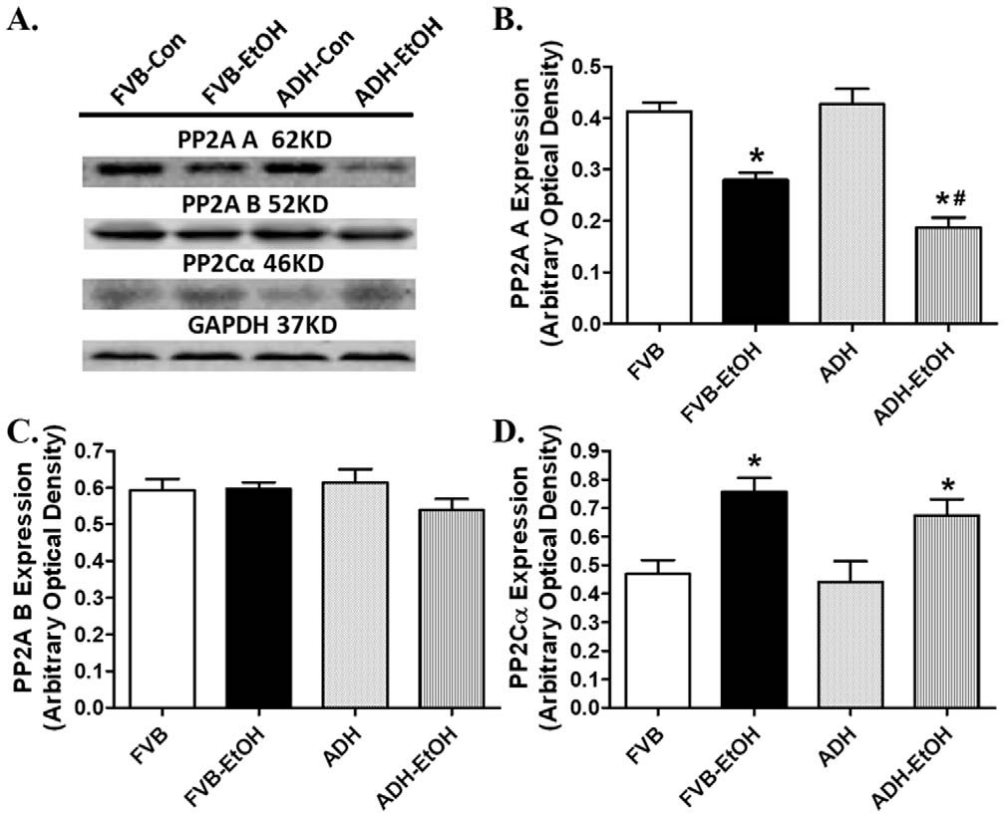
Expression of protein phosphatases in myocardium from FVB and ADH mice with or without acute ethanol challenge (3 g/kg, i.p. for 3 days). A: Representative gel blots depicting expression of PP2AA, PP2AB, PP2Cα and GAPDH (loading control); B: PP2AA; C: PP2AB; and D: PP2Cα. Mean ± SEM, n = 6–8 samples per group, * p<0.05 *vs.* FVB group, # p<0.05 *vs*. FVB-EtOH group.

To further explore the mechanism of action behind ADH and/or ethanol-induced response in AMPK activation, expression of protein phosphatase PP2A (both PP2AA and PP2AB) and PP2C was examined in FVB and ADH myocardium with or without acute ethanol challenge. Neither acute ethanol exposure nor ADH affected the expression of PP2AB. Interestingly, expression of PP2AA was significantly downregulated by acute ethanol exposure, the effect of which was intensified by ADH. To the contrary, expression of PP2Cα was upregulated by acute ethanol challenge, the effect was unaffected by ADH transgene. In addition, ADH transgene itself did not affect the expression of PP2AA and PP2Cα (Fig. 9).

### Effect of AMPK inhibition on acute ethanol exposure-induced changes in cell shortening

**Figure 10:**
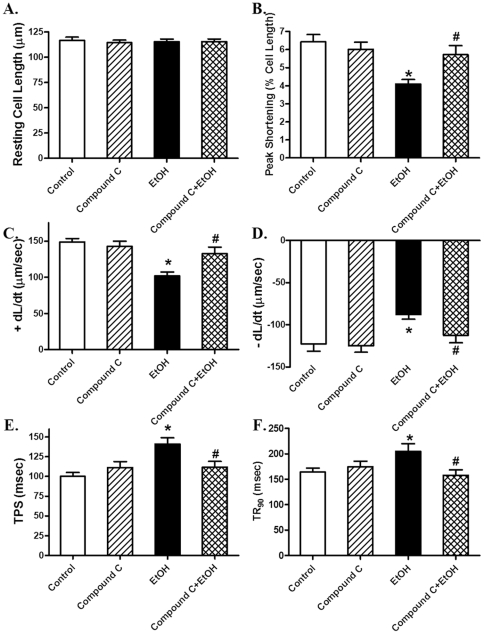
Effect of the AMPK inhibitor compound C on ethanol-induced cardiomyocyte contractile defects. Freshly isolated cardiomyocytes from FVB mice were incubated with ethanol (240 mg/dl) in the presence or absence of compound C (10 µM) for 2 hrs. A: Resting cell length; B: Peak shortening (normalized to resting cell length); C: Maximal velocity of shortening (+ dL/dt); D: Maximal velocity of relengthening (− dL/dt); E: Time-to-peak shortening (TPS) and F: Time-to-90% relengthening (TR_90_). Mean ± SEM, n = 59–77 cells from 3 mice per group, * p<0.05 *vs.* control group, # p<0.05 *vs.* ethanol (EtOH) group.

To further examine the role of AMPK in ethanol-induced cardiac contractile defects, freshly isolated cardiomyocytes from wild-type FVB mice were treated with ethanol (240 mg/dl) for 2 hrs in the absence or presence of the AMPK inhibitor compound C (10 µM) [19]. Our data shown in Fig. 10 indicated that the short-term treatment of ethanol significantly decreased PS and ± dL/dt as well as prolonged TPS and TR_90_ without affecting resting cell length. Interestingly, compound C significantly alleviated the acute ethanol exposure-induced mechanical defects without eliciting any effects on cardiomyocyte mechanics by itself. These data favor a likely role of AMPK in the acute ethanol exposure-induced cardiac contractile dysfunction.

## Discussion

Alcoholic cardiomyopathy is featured by compromised myocardial contractility [2], [3], [4], [7]. This is coincided with our current observation of reduced myocardial contraction in ethanol-challenged murine hearts. Furthermore, data from our present study revealed change in the AMP-to-ATP ratio and hyperactivated AMPK signaling cascade following acute ethanol challenge, which is associated with ethanol-elicited cardiac dysfunction, intracellular Ca^2+^ mishandling, glucose intolerance and hyperinsulinemia. More importantly, our study provided evidence for the first time that the cellular fuel AMPK signaling cascade following ethanol exposure may be augmented by ADH. These observations are in favor of the notion that facilitated ethanol metabolism via ADH enzyme exacerbates acute ethanol toxicity-induced myocardial dysfunction and glucose intolerance possibly related to over-stimulation of the cellular fuel AMPK.

Results from our study revealed that ADH accentuated ethanol-induced cardiac contractile and intracellular Ca^2+^ anomalies. This is supported by results from our current study in that ADH elicited a more pronounced deterioration in the heart contractile function, as evidenced by reduced ± dP/dt and LVDP, depressed peak shortening and ± dL/dt associated with prolonged TR_90_ following ethanol challenge Moreover, ADH deteriorated ethanol-induced decrease in electrically-stimulated rise in intracellular Ca^2+^, prolongation of intracellular Ca^2+^ decay rate and loss of intracellular Ca^2+^ cycling/handling capacity (the steeper staircase in peak shortening change in response to increased stimulus frequency). These data are in agreement with our earlier observations following alcohol intake using similar transgenic murine model [13], [15]. More interestingly, the ADH and/or acute ethanol-induced cardiac contractile and intracellular Ca^2+^ responses were coordinated with hyperactivated AMPK signaling cascade including phosphorylation of AMPK, ACC and LKB1 as well as downregulation of protein phosphatase PP2A subunit and PPAR-γ. The serine/threonine protein kinase AMPK is a cellular energy sensor for glucose and lipid metabolism regulating the cellular energy balance. The heterotrimeric AMPK enzyme is widely expressed in adipose tissue, skeletal muscle, liver, heart, pancreas and brain [20], [21]. Physiological stimuli such as ischemia reperfusion, hormones and nutrients may activate AMPK by elevating intracellular AMP/ATP ratio. Data from our current study revealed an elevation in cardiac AMP-to-ATP ratio following acute ethanol challenge with a further raise in the ADH ethanol-treated mice. Typically, elevation of intracellular AMP or the AMP-to-ATP ratio serves as the main activator of AMPK through several mechanisms. AMP itself is known to directly turn on AMPK. Second, AMP activates the AMPK upstream kinase LKB1 to phosphorylate α-subunit of AMPK at Thr172. Last, the binding of AMP to AMPK renders it a better substrate for LKB1 and reduces its substrate affinity for protein phosphatase [22], [23], [24]. The AMPK upstream kinase LKB1 kinase monitors the levels of glucose and the AMP/ATP ratio, governing the Thr172 phosphorylation of the α catalytic subunit of AMPK [22], [25]. Result from our current study revealed that acute ethanol treatment turned on LKB1 phosphorylation with a further increase in ADH mice, supporting a likely role of LKB1 in the ADH and ethanol-induced AMPK activation (shown by phosphorylation of AMPK and ACC). AMPK activation limits biosynthetic pathways while facilitating catabolic pathways to conserve energy by ATP generation through enhancing oxidative metabolism and mitochondrial biogenesis [26], [27]. This is somewhat supported by our current data of upregulated PGC-1α expression in response to acute ethanol exposure although such effect was unaffected by ADH overexpression. Although data from our present study fail to provide any precise mechanism of action behind the hyperphosphorylated LKB1/AMPK signaling cascade, accumulation of reactive oxygen species in response to ethanol exposure is believed to play an important role. Ethanol or acetaldehyde has been shown to trigger oxidative stress and apoptosis via activation of stress signaling such as c-Jun phosphorylation [1]–[3]. This is supported by our recent findings of elevated O_2_^−^ anion production and apoptosis in murine hearts following ethanol challenge, the effect of which was accentuated by the ADH transgene [13]. Evidence from our group also revealed that ADH produced greater levels of lipid peroxidation and protein carbonyls in hearts from the alcohol-fed mice [4], consolidating an likely role of free radical formation in AMPK activation [28] following acute ethanol exposure. Ethanol has been shown to elicit a number of pathophysiological responses including compromised glucose transport and gastric acid secretion via an AMPK-dependent manner [29], [30], similar to our current finding. Consistently, AMPK activity was found elevated within a few hours after ethanol exposure in liver and skeletal cells [31], [32]. This is also supported by our observation that the AMPK inhibitor compound C effectively alleviated acute ethanol exposure-induced cardiomyocyte dysfunction. Nonetheless, recent finding from Crabb and colleagues suggested that ethanol is capable of inhibiting (rather than activating) hydrogen peroxide-induced AMPK activation in hepatoma cells [23], suggesting a likely paradoxical role of AMPK in ethanol-elicited effects. It is possible that difference in tissue type and experimental setting (*in vivo* treatment versus cell culture) may contribute to the discrepant effect of ethanol on AMPK activation between our study and the previous report [23].

Observation from our study also depicted downregulation of PP2A (PP2AA in particular without change in PP2AB) following acute ethanol challenge, the effect of which was augmented by the ADH transgene. To the contrary, acute ethanol exposure elicited a comparable upregulation of PP2Cα in both FVB and ADH mice. PP2A and PP2Cα, two protein phosphotases, are known to dephosphorylate and inactivate AMPK [23]. AMP was reported to increase AMPK activation by directly inhibiting phosphatase or indirectly making AMPK a poor substrate for phosphatases [33]. Therefore, ADH and ethanol-induced effect on protein phosphatases (especially PP2A) may play a role in hyperactivated AMPK signaling in our current experimental setting. Our findings suggest that the phosphorylation of AMPK at Thr172 may be influenced by the expression of PP2A but less likely PP2Cα in ethanol-treated ADH mice.

AMPK plays an essential role for the maintenance of myocardial function [34]. Interrupted AMPK signaling integrity may contribute to the onset and development of neurodegenerative disorders, diabetes and ischemia reperfusion-induced heart damage [34]. Recently, AMPK signaling defect also received some attentions in alcoholic complications since ethanol metabolism directly affects energy metabolism such as reduced ATP level and mitochondrial respiratory rates [11]. This is consistent with our current finding of elevated phosphorylation of AMPKα, ACC and LKB1 associated with enhanced AMP-to-ATP ratio in hearts following acute ethanol exposure. More intriguingly, the ethanol-induced changes in AMPK, ACC, LKB1 and the AMP/ATP ratio were exaggerated by ADH, consolidating the contribution of ethanol metabolism to energy metabolism. These observations were in line with the changes in myocardial contractility and oral glucose tolerance in FVB and ADH mice following ethanol exposure, suggesting a likely role of AMPK in ADH-induced exacerbation of myocardial injury in response to ethanol exposure.

Insulin sensitivity plays an essential role in myocardial pathology including ischemia and reperfusion injury, diabetic and alcoholic cardiomyopathy [22], [34]. Insulin also regulates various aspects of cardiovascular metabolism and function including glucose and long-chain fatty acid metabolism, protein translation and vascular tone [35]. As mentioned earlier, AMPK signaling pathway is involved in insulin signaling in the heart [36], [37]. In our study, the over-phosphorylated AMPK signaling following facilitated ethanol metabolism (ADH) may contribute to dampened insulin signaling following ethanol challenge at a checkpoint underneath the insulin receptor. Our observation of equally down-regulated insulin receptor in the ethanol-treated FVB and ADH mice does not favor a major role of insulin receptor in the ADH-accentuated myocardial mechanical defects. The fact that ADH augmented acute ethanol exposure-induced downregulation in PPAR-γ substantiated the critical role of post-insulin receptor signaling in the ethanol-induced cardiac contractile defect and intracellular Ca^2+^ mishandling. PPAR-γ has been shown to play a critical role in cardiac contractile function. PGC-1α, a member of a family of transcription coactivators, is essential for mitochondrial biogenesis and participates in the regulation of both carbohydrate and lipid metabolism [38]. Our finding of comparable upregulation in the PGC-1α expression or unchanged Glut4 levels does not seem to favor an involvement of mitochondrial biogenesis and Gut4 in the ADH-exacerbated myocardial defect following acute ethanol challenge. Although long-term ethanol exposure is known to inhibit Glut4 expression via an AMPK-dependent pathway in adipocytes [29], our current data were unable to address the intimate interplay among AMPK signaling, insulin sensitivity, myocardial and intracellular Ca^2+^ derangement following acute ethanol exposure.

In summary, the present study has provided convincing evidence that cardiac overexpression of ADH exacerbated acute ethanol exposure-induced myocardial contractile and intracellular Ca^2+^ dysregulation associated with altered insulin sensitivity and hyperphosphorylated AMPK signaling. These data support a possible role of ethanol metabolism and AMPK signaling cascade in acute ethanol toxicity-elicited alcoholic myopathic alteration. These data should shed some lights towards a better understanding of the role of cardiac energy metabolism, AMPK and insulin signaling in alcoholic myocardial dysfunction. On the other hand, pharmacological inhibition of ADH enzyme has been shown to diminish or prevent ethanol-induced undesirable effects such as lactacidemia and hypoglycemia [39], indicating the therapeutic potential of this crucial ethanol oxidation enzyme. Further investigation is warranted to better elucidate the therapeutic value of AMPK cascade in the clinical management of binge drinking-associated myopathic anomalies.
